# Solvated interaction energy: from small-molecule to antibody drug design

**DOI:** 10.3389/fmolb.2023.1210576

**Published:** 2023-06-07

**Authors:** Enrico O. Purisima, Christopher R. Corbeil, Francis Gaudreault, Wanlei Wei, Christophe Deprez, Traian Sulea

**Affiliations:** Human Health Therapeutics Research Centre, National Research Council Canada, Montreal, QC, Canada

**Keywords:** binding affinity, solvation, protein-ligand interaction, scoring function, antibody affinity maturation

## Abstract

Scoring functions are ubiquitous in structure-based drug design as an aid to predicting binding modes and estimating binding affinities. Ideally, a scoring function should be broadly applicable, obviating the need to recalibrate and refit its parameters for every new target and class of ligands. Traditionally, drugs have been small molecules, but in recent years biologics, particularly antibodies, have become an increasingly important if not dominant class of therapeutics. This makes the goal of having a transferable scoring function, i.e., one that spans the range of small-molecule to protein ligands, even more challenging. One such broadly applicable scoring function is the Solvated Interaction Energy (SIE), which has been developed and applied in our lab for the last 15 years, leading to several important applications. This physics-based method arose from efforts to understand the physics governing binding events, with particular care given to the role played by solvation. SIE has been used by us and many independent labs worldwide for virtual screening and discovery of novel small-molecule binders or optimization of known drugs. Moreover, without any retraining, it is found to be transferrable to predictions of antibody-antigen relative binding affinities and as accurate as functions trained on protein-protein binding affinities. SIE has been incorporated in conjunction with other scoring functions into ADAPT (Assisted Design of Antibody and Protein Therapeutics), our platform for affinity modulation of antibodies. Application of ADAPT resulted in the optimization of several antibodies with 10-to-100-fold improvements in binding affinity. Further applications included broadening the specificity of a single-domain antibody to be cross-reactive with virus variants of both SARS-CoV-1 and SARS-CoV-2, and the design of safer antibodies by engineering of a pH switch to make them more selective towards acidic tumors while sparing normal tissues at physiological pH.

## 1 Introduction

Structure-based drug design depends on computational methods for predicting binding modes and estimating binding affinities. These typically rely on scoring functions that can be classified into four categories: empirical, knowledge-based, physics-based and, more recently, artificial intelligence-based, encompassing descriptor-based machine learning to deep learning approaches (Gohlke and Klebe, 2002; Gilson and Zhou, 2007; Liu and Wang, 2015; Geng et al., 2019; Dhakal et al., 2022). For the last 15 years, our group has been developing and applying Solvated Interaction Energy (SIE), a structure-based scoring function for predicting intermolecular binding affinities in aqueous solution (Naim et al., 2007; Cui et al., 2008; Sulea et al., 2011; Sulea and Purisima, 2012b; Sulea et al., 2016; Vivcharuk et al., 2017). Falling within the physics-based category, scoring functions such as SIE continue to be attractive as their results are more readily interpretable due to the connection to the underlying physics enabling a rationally-driven modulation of binding affinities. As its name suggests, solvation is an important component of SIE given its major role in binding and is captured through a continuum solvation model.

In this overview, we describe the development of the SIE scoring function and its applications. A notable characteristic of this scoring function is its versatility. Although initially developed in the context of predicting binding affinities for protein-ligand interactions involving small molecules, SIE has found much broader applicability. We give examples of this versatility as we survey applications to docking and virtual screening for both small-molecule ligands and biologics. A demanding test of the usefulness and performance of any scoring function is its application to a wide variety of biological systems as well as its use in the hands of users from other laboratories aside from that of the developers. To assess those, we analyzed the usage and performance of SIE as reported in close to 400 publications.

## 2 The SIE scoring function approach

### 2.1 Original calibration of SIE

The overarching theme for the development of the SIE scoring function was to leverage existing work in force fields while keeping the number of fitted parameters to a minimum to guard against overfitting. Leveraging the works of AMBER (Case et al., 2005; Hornak et al., 2006) and GAFF (Wang et al., 2004) for the van der Waals and electrostatics interactions allowed SIE to use well-established sets of basic parameters for these terms. In addition, solvation terms from a continuum solvation model were supplemented. The linear combination of these terms gave rise to a functional form of SIE with five parameters that were fitted to reproduce experimental binding affinities (Eq. 1) (Naim et al., 2007). It should be noted that only 3 parameters (*ρ, D*_in_ and *γ*) affect the actual correlation with experiment, e.g., the relative ranking of affinities.

$$\Delta G_{bind}\left(\rho ,D_{in},\alpha ,\gamma ,C\right)=\alpha \left[{E_{vdW}+E}_{coul}\left(D_{in}\right)+\Delta G_{bind}^{R}\left(\rho ,D_{in}\right)+\gamma \Delta MSA\left(\rho \right)\right]+C$$ (1)

*E*_vdW_ and *E*_coul_ are the intermolecular van der Waals and Coulomb energies using the AMBER/GAFF force field. The Coulomb energy depends on the parameter *D*_in_, the solute interior dielectric constant, as does the change in solvation reaction field energy, Δ*G* ^R^_bind_. The Δ*MSA* term is the change in molecular surface area, and its contribution to the nonpolar solvation energy is proportional to area with a coefficient of γ. Both Δ*G* ^R^_bind_ and Δ*MSA* depend on the choice of radii used to define the solute-solvent dielectric boundary. These are set to the AMBER/GAFF Lennard-Jones radii linearly scaled by a factor ρ, one of the fitting parameters. The coefficient α is a global scaling factor and *C* a translation constant to bring the SIE score to the same magnitude as the experimental values of a training set.

The five parameters were fitted by minimizing the mean absolute deviation of the predicted versus experimental binding free energies for 11 targets comprising 99 protein-ligand complexes (Naim et al., 2007). These targets had six or more representative protein-ligand crystal structures with corresponding published binding affinity data. The published fitted parameters in use since 2008 are *α* = 0.1048, *D*_in_ = 2.25, *ρ* = 1.1, *γ* = 0.0129 kcal/(mol·Å^2^) and *C* = −2.89 kcal/mol (Cui et al., 2008). Note that these are slightly modified from the original 2007 fitted parameters (Naim et al., 2007). Overall, the correlation yielded a mean absolute deviation of about 1.4 kcal/mol in binding free energy for the 99 complexes.

We noted that the global proportionality coefficient α significantly scales down the sum of the various energy terms. We interpreted this as roughly capturing entropy-enthalpy compensation (Sharp, 2001; Reynolds and Holloway, 2011), i.e., the more negative ΔH is, the greater the −TΔS cost of binding will be. Gilson and coworkers have also observed a strong correlation between changes in computed configurational entropy, ΔS, and changes in potential energy plus solvation, Δ(U + W), upon binding in their studies of host-guest complexes (Chang and Gilson, 2004; Chen et al., 2004). One can roughly think of this as the tendency of stronger interactions to narrow the energy well of the complex, increasing the entropic cost of binding. In their study, Gilson and coworkers found that the entropic cost, −TΔS, cancels out about 90% of Δ(U + W). That degree of compensation is similar to the scaling by α of the interaction energy plus solvation in SIE. Thus, although SIE does not explicitly include an entropy term (aside from the solvation entropy included in the nonpolar surface area term), some of the entropic effects are implicitly contained in the α scaling factor.

### 2.2 Incorporation of solvation models

The solvation contribution is a critical term in the SIE score. This is incorporated using a continuum solvation model with the electrostatic component calculated using a boundary element solution (Purisima and Nilar, 1995; Purisima, 1998) of the Poisson equation. Our implementation of the boundary element method (BEM) for continuum electrostatics was noteworthy in that it was one of the earliest implementations of BEM that was computationally efficient enough to be applied to macromolecular systems (Purisima and Nilar, 1995). The nonpolar contribution was proportional to the molecular surface area. Over time it was realized that the usual continuum solvation model based on a table of atomic radii for a set of atom types and the use of just a surface area term for all of the nonpolar contributions had serious deficiencies. In particular, it could not capture the observed charge asymmetry of reaction field energies (Purisima and Sulea, 2009). In addition, the simple surface area model missed some of the nuances of solute-solvent van der Waals interactions. To address these deficiencies, the FiSH (First Shell Hydration) solvation model was developed (Corbeil et al., 2010). This makes the Born radii dependent not just on the atom type but also on the local electrostatic potential felt at the dielectric boundary. In addition, a continuum van der Waals model was incorporated that had two terms, one for the interaction with the first shell of water around the solute, and a second one for the interaction with more distant water molecules. Both the standard and FiSH solvation models are available within the SIE program.

### 2.3 Single-structure and MD-trajectory modes

SIE can be applied to a single conformation of a complex or it can be applied to snapshots of a molecular dynamics (MD) simulation from which SIE averages are calculated (Cui et al., 2008). A software package, *sietraj*, consisting of scripts and executables used to process a single conformation or an AMBER-generated MD trajectory is downloadable from https://mm.nrc-cnrc.gc.ca/sietraj/ (Sulea and Purisima, 2012b). Virtual alanine scanning of selected residues using an MD trajectory can be done as well. Calculating an average SIE from snapshots of an MD trajectory allows sampling conformations around an energy minimum and has the potential advantage of reducing the bias that may come from a single static conformation. However, for virtual screening applications, scoring a single conformation from a well-prepared energy-minimized structure can give satisfactory results and sufficient enrichment.

### 2.4 Relation to other physics-based methods

The different physics-based scoring functions in use vary in terms of the van der Waals parameters, partial charges, treatment of solvation and conformational sampling. Most have a simplified solvation term for computational speed. Of the more commonly used ones, MM-PBSA (Massova and Kollman, 2000; Rastelli et al., 2010) is the most similar to SIE. In fact, the two methods are sometimes used side by side to corroborate the results of each method. Just like SIE, it uses the AMBER/GAFF force field for van der Waals parameters and partial charges. It also models solvation effects with a high-quality continuum solvation model. MM-PBSA does have the further ability to incorporate ionic strength, which SIE does not have. Another difference between the two methods is that MM-PBSA has an explicit entropic term based on a normal mode calculation, i.e., the curvature at the bottom of the multi-dimensional potential energy well of an energy-minimized snapshot. However, this can make it significantly more expensive than SIE when analyzing an MD trajectory. Interestingly, although MM-PBSA has a more detailed accounting of entropy, MM-PBSA greatly over-estimates the magnitude of the absolute binding free energy while SIE scores are typically closer in magnitude to the measured binding affinities.

Most of the computational time of SIE is spent on the boundary element solution (BEM) of the Poisson equation for the continuum electrostatics solvation model. The SIE score requires three calculations–the solvation free energy of the complex and of each partner in the free state. As an example of computational cost, for a complex of two proteins each about 120 residues, these three calculations altogether take about 8 CPU seconds on a single core (Intel Xeon Silver 4116).

More detailed but computationally expensive physics-based methods are alchemical free energy methods (Chodera et al., 2011; Mey et al., 2020). These methods calculate the relative free energy change by gradually transforming one molecular entity into another and require extensive molecular dynamics sampling. Although in principle more rigorous than end-point methods such as SIE, in the blind tests (Skillman, 2012; Muddana et al., 2014; Gaieb et al., 2018) of predicting binding affinities mentioned in Section 3.2, they have not shown a clear advantage over the simpler end-point methods in terms of accuracy.

## 3 Small-molecule drug design

Intended for applications in small-molecule drug design, the original training of the SIE function was carried on small-molecule–protein binding data. The robustness of the trained SIE function in terms of accuracy and transferability was demonstrated by its many prospective and retrospective applications in laboratories worldwide, compiled and analyzed in Section 5. We also unbiasedly tested and benchmarked SIE during the past 15 years in community-wide challenges, including SAMPL (Statistical Assessment of the Modeling of Proteins and Ligands; https://www.samplchallenges.org/), CSAR (Community Structure-Activity Resource; http://www.csardock.org/) and D3R-GR (Drug Design Data Resource–Grand Challenge; https://drugdesigndata.org/about/grand-challenge) (Table 1). We not only tested SIE’s accuracy to predict binding affinities, but also its underlying solvation models as well as its derivative applications to ligand docking and virtual screening.

**TABLE 1:** Testing of SIE and related methods in community-wide challenges.

Challenge	Scope of SIE prediction evaluation	Small-molecule binding target
SAMPL-1	Solvation^(a)^	c-Jun N-terminal kinase 3 (JNK3)
	Binding affinity^(b)^	
SAMPL-2	Solvation^(c)^	N/A
SAMPL-3	Solvation^(d)^	Trypsin
	Binding affinity^(b)^	Cucurbit [*n*]uril hosts
	Virtual screening^(b)^	
SAMPL-4	Binding affinity^(e)^; ;	HIV-integrase (HIV-I)
	Docking^(e)^	Cucurbit [*n*]uril and OctaAcid hosts
	Virtual screening^(e)^	
CSAR-2010	Binding affinity^(f)^	>200 different proteins
CSAR-2013/14	Binding affinity^(g)^	Steroid-binding protein (SBP)
	Docking^(g)^	Factor Xa (FXA)
		Spleen tyrosine kinase (Syk)
		tRNA guanine-methyltransferase (TrmD)
D3R-GC2	Binding affinity^(h)^	Farnesoid X receptor (FXR)
	Docking^(h)^	

(a) Sulea et al. (2009), (b) Sulea et al. (2012), (c) Purisima et al. (2010), (d) Sulea and Purisima (2012a), (e) Hogues et al. (2014), (f) Sulea et al. (2011), (g) Hogues et al. (2016), (h) Hogues et al. (2018b).

### 3.1 Solvation free energy

The prediction accuracy and transferability of the BEM and FiSH solvation models underpinning SIE was tested early on. The first stringent test was predicting hydration free energies on the challenging drug-like SAMPL-1 blind data set of 63 highly polyfunctional organic compounds (Sulea et al., 2009). Surprisingly, we found that the BEM electrostatic-only solvation model afforded smaller absolute errors and was more transferable than a more complex continuum electrostatics-dispersion (CED) solvation model that added continuum nonpolar solvation terms parametrized by atom types. Indeed, even if the prospective predictions of the more complex CED model were highly correlated to experiment (*R* ^2^ = 0.82), they had systematic errors, mainly associated to compounds with multiple hydrogen bonds. This apparent shortcoming prompted the evolution of the CED solvation model into the FiSH solvation model, by separating the first shell of hydration and parametrizing both electrostatic and non-electrostatic terms based on explicit-solvent simulation data (Corbeil et al., 2010; Sulea et al., 2010). Testing of the FiSH model on the obscured set of 23 highly diverse and polyfunctional compounds of the SAMPL-2 challenge indicated improved accuracy and transferability relative to its CED predecessor model, with mean unsigned errors (MUE) more consistently below 2 kcal/mol (Purisima et al., 2010). However, accuracy was still varied across functional classes, calling for more detailed parametrization zooming on particular chemical classes and polyfunctional compounds. This was demonstrated in SAMPL-3, which challenged solvation predictors with 36 poly-chlorinated analogs (Sulea and Purisima, 2012a). A stark difference was observed between the accuracies of FiSH solvation predictions for aliphatic poly-Cl (*R* ^2^ = 0.52; MUE = 0.66 kcal/mol) and aromatic poly-Cl (*R* ^2^ = 0.05; MUE = 3.43 kcal/mol) compounds. Recalibration of the aromatic Cl parameters on explicit-solvent simulation data improved FiSH model predictions.

### 3.2 Binding affinity

Most prospective testing of SIE was dedicated to binding affinity predictions. In its first blind test (SAMPL-1), the original standard SIE parametrization achieved reasonable predictions (*R* ^2^ = 0.36; MUE = 0.92 kcal/mol) on the JNK3 data set consisting of 49 diverse ligands, each with its own co-crystal structure with the kinase, in addition to 10 docked models of known inactive analogs (Sulea et al., 2012). Absolute binding affinities were also predicted within the actual range while the inactives were separated reasonably well from the actives, indicating applicability to virtual screening.

SIE underwent stringent testing in SAMPL-3/4 (Skillman, 2012; Muddana et al., 2014), which blindly challenged the SIE’s applicability domain with three extreme scenarios: i) weak-affinity fragment-sized ligands binding to a protein target (trypsin); ii) high-affinity guest ligands binding to small targets (hosts or cages); and iii) ligands exhibiting a very narrow dynamic range below 2 kcal/mol for binding to a protein target (HIV-I) (Sulea et al., 2012; Hogues et al., 2014). Importantly, affinity predictions were made on computationally docked ligands, for which the Wilma-SIE method (Section 3.3) was employed. SIE provided affinity predictions with an MUE of 2.24 kcal/mol for the trypsin–fragments set, which were significantly improved by incorporating the newer FiSH solvation model (MUE 0.98 kcal/mol). This was also found on in the HIV-I set. SIE predictions in the host-guest systems were acceptable (*R* ^2^ 0.5–0.7) but suffered from an overestimation in absolute terms, which was corrected by rescaling the entropy-related factor, α, which may depend on the rigidity of the target molecule. Even with experimentally solved binding modes, SIE predictions lacked correlation with experimental affinities in the HIV-I set having binding affinities within 2 kcal/mol range; nonetheless, SIE was able to correctly signal the narrowness of the data range. Another conclusion from the HIV-I test set was that using a common protein structure for all ligands can reduce the noise.

SIE testing in the CSAR-2013/14 (Carlson, 2016) and D3R-GC2 (Gaieb et al., 2018) blind challenges allowed assessment of performance and transferability across a wider range of protein systems representative of real-life applications (Hogues et al., 2016; Hogues et al., 2018b). Affinity ranking of congeneric ligands after cross-docking was reasonably achieved in the SBP, Syk and TrmD systems, with Spearman rank-order correlation coefficients (S) ∼0.6. Poor ranking of FXA ligands was possibly due to protein domains not included in the calculations. Ligand preparation in the Syk set underscored the critical role of correct assignment of protonation states to the SIE performance. Including the FiSH model improved cross-docking but worsened affinity predictions, which pointed to a need for further fine-tuning of this newer solvation model. The FRX set from the D3R-GC2 blind challenge posed the formidable task of predicting ligand binding affinity to a highly flexible receptor. A possible cause for the difficulty of SIE function to predict binding affinities in this scenario is the internal energy strain arising from conformational differences in the receptor across complexes, which may need to be properly incorporated into SIE for flexible targets.

The most extensive testing of SIE purely for affinity prediction was done on the CSAR-2010 scoring set (Dunbar et al., 2011) consisting of high-resolution co-crystal structures for 343 protein-ligand complexes with high-quality binding affinity data spanning 18 kcal/mol and highly diverse protein targets (Sulea et al., 2011). SIE predicted binding affinities for the curated CSAR-NRC-HiQ dataset that were well in the range of experimental values (MUE = 1.98 kcal/mol; *R* ^2^ = 0.38). Predictions were found to be very sensitive to the assignment of protonation and tautomeric states in the complex, and to the treatment of metal ions near the protein-ligand interface. Retraining of the SIE function on this large and diverse set gave marginal improvements with small changes in optimal parameters and was not warranted.

### 3.3 Docking

A natural extension of the SIE function is docking, i.e., ranking binding modes (poses) of a ligand to a protein target. To this end, SIE was integrated into the exhaustive docking program Wilma (Sulea et al., 2012; Hogues et al., 2014; Hogues et al., 2016). Briefly, Wilma uses a brute-force, exhaustive searching approach where interaction modes with the rigid protein of all the discrete rotational and translational states of ligand conformations are enumerated, scored, clustered and ranked using a simple fast-scoring function. A few hundreds top-ranked poses produced by Wilma are then energy-minimized and rescored by SIE. The goal is to provide an accurate docking solution as the top-1 SIE-scored pose. The same SIE parametrization used for affinity prediction is also employed for docking, allowing consistency between pose ranking for each ligand and affinity ranking between different ligands.

Extensive testing of Wilma-SIE indicated that ligand docking can be achieved with high accuracy and is an easier task than binding affinity scoring. The power of Wilma-SIE in pose selection and cross-docking against multiple targets and ligand classes was unequivocally demonstrated in the CSAR-2013/14 blind challenge (Hogues et al., 2016). In all 24 pose-selection tests on 4 different protein targets, Wilma-SIE ranked the native pose as best among carefully generated sets of decoy conformations. Large score separations of native poses indicated robustness in pose scoring. Cross-dockings were also accomplished with high accuracies for various systems, with ligand median RMSD (mRMSD) values around 1 Å from the crystal structures. Both Wilma-SIE and Wilma-SIE + FiSH generated docking predictions among the best-performing submissions. In terms of consistency and system transferability, they were the only submissions with mRMSDs below 1.5 Å on every system. This level of performance was for top-1 poses ranked by SIE or SIE + FiSH over multiple target conformations. Using SIE + FiSH for pose scoring lead to somewhat better docking accuracy overall as well as for individual targets, with mRMSD of 0.6 Å by Wilma-SIE + FiSH in the Syk set.

Although the optimal regime of Wilma-SIE is for high-affinity ligands with low-to-moderate flexibility, the SAMPL-4 blind test on the HIV-I set demonstrated that Wilma-SIE can sometimes dock accurately even weak-affinity ligands (*K*_D_ > 0.1 mM) with high flexibility (>8 rotatable bonds) (Hogues et al., 2014). In D3R-GC2, the rigid-protein docking method Wilma-SIE faced the FXR target that exhibits significant backbone movement in response to ligand binding (Hogues et al., 2018b). Use of the conformational ensembles from publicly available structures of FXR allowed Wilma-SIE to predict poses with mRMSD of 1.4 Å on the set of 36 FXR diverse ligands, and rank amongst the best pose-prediction methods of the challenge. However, the success rate would have been much lower if only a single structure were used.

### 3.4 Virtual screening

With excellent scoring abilities for both binding affinity and pose selection, one of the most practical applications of SIE is virtual screening of compound libraries against a given target protein structure. Typical libraries of available multi-million drug-like compounds, e.g., ZINC (Sterling and Irwin, 2015), can be efficiently processed by Wilma-SIE, which is fully scalable and parallelizable on available computational resources. The first assessment of the SIE performance in virtual screening indicated excellent enrichments of true actives within decoy sets for estrogen receptor and thymidine kinase as screening targets, particularly in the latter more challenging system having weak binding affinities for the true binders (Naim et al., 2007). Wilma-SIE can be used for screening of not only drug-like but also fragment-like ligands, as demonstrated in the SAMPL-3 blind challenge on trypsin screening, where it achieved a good enrichment of the 20 true actives amongst 500 fragment-like ligand library with an AUC-ROC of ∼0.7 (Sulea et al., 2012). The early enrichment performance was particularly good, with 50% of true actives recovered with false-positive rates of 15% for Wilma-SIE and 3% for Wilma-SIE + FiSH. The SAMPL-4 blind test showed that Wilma-SIE is not suited for detection of promiscuous weak and flexible ligands, although even in such difficult cases it can lead to better-than-random virtual screening results (Hogues et al., 2014).

## 4 Biotherapeutics design

### 4.1 Antibody-antigen affinity ranking

With the rise of monoclonal antibodies (mAbs) as a promising class of biotherapeutics, the SIE function was evaluated for its ability to predict protein-protein binding affinities. An immediate application is antibody optimization, with a long-term goal towards *de novo* protein engineering. A first study assessed the transferability of the original SIE parametrization to predict changes in antibody-antigen binding affinities (Sulea et al., 2016). To this end, we assembled Single-Point Mutant Antibody Binding (SiPMAB), a dataset of 212 antibody mutants from 7 systems having high-resolution crystal structures of parental antibodies and high-quality binding affinity measurements. The SIE function coupled with a protocol limited to sampling only the mutated side chain was able to reasonably predict relative binding affinities (S ∼0.6) without any reparameterization of the original SIE function trained on small-molecule binding. Binding affinity ranking performance was maintained for each of the 7 systems and other subsets including non-alanine and charge-altering mutations. Performance was further enhanced using consensus ranking over multiple scoring functions alongside SIE, such as FoldX (Guerois et al., 2002) and Rosetta (Kortemme and Baker, 2002; Ó Conchúir et al., 2015). The consensus scores were obtained by converting the scores from the various scoring functions into normalized z-scores and producing an average z-score for each mutant. This facilitated combining the disparate magnitudes and scales of the different scoring functions.

### 4.2 Affinity maturation of antibodies

Traditional experimental approaches such as library display and screening are incapable of thoroughly exploring the vast mutational space available to a typical antibody complementarity determining region (CDR; ∼60 residues). One meaningful way to systematically explore and prioritize this space is to examine single-point mutants first and then combine validated hot spots into multiple-point mutants. A cost-effective protocol is to leverage the speed of *in silico* screening followed by experimental validation of only a small number of predicted hits in each mutation round. To this end, we developed Assisted Design of Antibody and Protein Therapeutics (ADAPT), a platform that interleaves structure-based virtual screening mutagenesis with experimental testing in order to optimize the binding affinity of a biologic (antibody) to its target (antigen) (Vivcharuk et al., 2017). The ADAPT-based affinity maturation eliminates false-positive predictions in two ways: i) early experimental validation of top-scored virtual hits, mainly at the single mutation stage, and ii) consensus affinity scoring over SIE and other popular scoring functions like FoldX and Rosetta. The platform is also designed to preserve protein folding upon mutation by using a computational filter.

Prospective applications of ADAPT affinity maturation in real-life projects (Table 2) led to 10–100-fold improvements in the dissociation constant (*K*_D_) for several Fab fragments of mAbs that originally bound their antigens with 0.05–50 nM affinities (Vivcharuk et al., 2017). ADAPT has also been applied successfully to improve the binding affinity of a single-domain antibody (sdAb) against *Clostridium difficile* toxin A by 10-fold, which led to improved functional efficacy and thermal stability of the optimized sdAb (Sulea et al., 2018). To achieve affinity improvements via ADAPT, only about 30–50 single to triple mutants need to be recombinantly produced and tested, a significant reduction of the aforementioned available mutational space.

**TABLE 2:** Prospective applications of SIE via ADAPT to antibody engineering.

Disease	Antigen	Binding modulation	Functional outcome
Cancer	Her2	10-fold *K*_D_ improvement^(a)^	Increased tumor growth inhibition; increased cellular internalization^(b)^
		1000-fold *K*_D_ range modulation^(b)^	Increased therapeutic window for cytotoxic ADCs^(b)^
		25-fold selectivity improvement towards acidic pH^(c)^	Inhibition of tumor spheroid growth only at acidic pH; widened therapeutic index^(c)^
	VEGF-A	100-fold *K*_D_ improvement^(a)^	—
	CD47	100-fold *K*_D_ improvement^(d)^	—
Infection	*C. difficile* Toxin A	10-fold *K*_D_ improvement^(e)^	Increased neutralization of cytotoxicity in cell culture^(e)^
	SARS-CoV-2 Spike variants	20–1000-fold *k*_off_ improvement for bivalent constructs^(f)^	Increased neutralization of infection in cell culture and in animal model^(f)^

(a) Vivcharuk et al. (2017), (b) Zwaagstra et al. (2019), (c) Sulea et al. (2020), (d) Cheng et al. (2019), (e) Sulea et al. (2018), (f) Sulea et al. (2022).

The utility of the consensus score is highlighted in Table 2 of Vivcharuk et al. (2017). For the bH1-VEGF system, only SIE had a good z-score for the G99D mutation. FoldX and Rosetta scored it poorly and ranked it 522 and 725, respectively. Due to SIE, the consensus z-score of this single mutant brought it within the top 50 consensus z-scores, making it to the list for experimental validation. In the bH1-HER2 system, only FoldX scored the I29R mutant well. SIE and Rosetta z-scores had them at ranks 107 and 153, respectively. However, the consensus z-score made it within the top 50 cutoff. In the Herceptin-HER2 system, only Rosetta had a good z-score for D102F, while the FoldX and SIE z-scores ranked it at 88 and 51, respectively. Again, the consensus z-score brought it within the cutoff for experimental consideration. All of these single mutants turned out to be components of the best triple mutants for their respective systems. They would have been missed for one or more systems had we relied on a single scoring function. The consensus scoring approach was also shown to be superior to each individual component scoring function on the SiPMAB data set in terms of AUC-ROC (Sulea et al., 2016).

In certain cases, it is beneficial to controllably weaken binding affinity, for example, to reduce toxicity of antibody-drug conjugates (ADCs) used in oncology. This approach takes advantage of the bivalency of mAbs and higher expression of antigens on tumor versus normal cells. ADAPT and SIE were employed to design a set of antibody mutants that evenly modulate binding affinity within a 4 kcal/mol range leading to identification of optimal ADCs with improved therapeutic windows (Zwaagstra et al., 2019).

ADAPT was used to broaden the specificity of an anti-SARS-CoV-1 sdAb that had only weak cross-reactivity with SARS-CoV-2 (Sulea et al., 2022). By applying ADAPT with the constraint of dual-affinity optimization simultaneously against coronaviruses from distinct phylogenetic clades, optimized sdAbs were found that neutralized the major variants of concern within the SARS-CoV-2 clade with superior pan-specificity and potency relative to the parental antibody.

A requirement for ADAPT is the availability of 3D structural data for the protein-protein interface subjected to affinity optimization, ideally from crystallographic experiments. Encouragingly, a recent study reports ADAPT-based affinity maturation of a weak-affinity anti-CD47 sdAb (*K*_D_ of 278 nM) by 87-fold based on an antigen-bound sdAb structure derived by homology modeling, molecular dynamics and protein-protein docking (Cheng et al., 2019).

### 4.3 Engineering pH-sensitive antibodies

For therapeutic applications in oncology, specific binding under the slightly acidic pH of solid tumors can reduce off-tumor binding and toxicity on normal cells living under physiological pH. Given that the average pKa of histidine in proteins is ∼6.4, virtual His screening of antibody CDR appears as a suitable approach to achieve pH-selective antigen engagement. To do this in ADAPT, SIE calculations are performed twice for each His mutant, in the protonated and neutral states, and then referenced the parental complex in both pHs. This approach was successfully applied to introduce pH-dependent binding in a variant of trastuzumab (Herceptin) binding the Her2 antigen overexpressed in breast cancer (Sulea et al., 2020). Designed antibody His mutants bound stronger to acidic cancer cells than to normal cells under physiological pH, and inhibited cell growth under acidic pH but not under physiological pH. In contrast, the parental antibody impacted tumor and normal cells similarly. In a larger-scale application, ADAPT and SIE could be used to retrofit the entire anticancer pipeline of antibodies with available 3D structures in complex with their onco-antigens (Wei et al., 2022). ADAPT could be similarly employed to engineer pH selectivity in the opposite direction for the design of recycling antibodies. In this scenario, overexpressed targets captured under physiological pH can be degraded more readily if they disengage from recycling antibodies in the slightly acidic endosomes inside the cells.

## 5 SIE in the wild

A literature analysis involving manual inspection of 377 distinct citations of 15 key methodological papers on SIE (Naim et al., 2007; Cui et al., 2008; Sulea et al., 2011; Sulea and Purisima, 2012b; Purisima and Hogues, 2012; Sulea et al., 2012; Hogues et al., 2014; Henry et al., 2016; Hogues et al., 2016; Sulea et al., 2016; Vivcharuk et al., 2017; Hogues et al., 2018a; Hogues et al., 2018b; Sulea et al., 2018; Sulea et al., 2020) (as of 21 December 2021) was undertaken (Supplementary Table S1). Only 36 of those citations are self-citations (10%). 50 of the 377 citations (13%) are for a different scoring function, called GBVI/WSA (Corbeil et al., 2012), calibrated on the SIE training dataset (Supplementary Table S2) and having a formalism similar to SIE but differing in its surface area calculations and force-field used. SIE has been used by hundreds of scientific groups around the globe (Supplementary Figure S1) with nearly 29% of citations from North-America, 31% from Asia, and 26% from Europe.

This literature was scrutinized to determine the use of SIE in research. In almost half of citations, SIE was used either prospectively (40 articles) to design new molecules or retrospectively (146 articles) to rationalize binding or a biological process. Predicted binding affinities were collected for instances in which experimental binding affinities were also reported. In total, two subsets of 275 and 150 data points for small-molecule and antibody complexes were collected from 65 to 5 articles, respectively (Supplementary Tables S3-S4). The small-molecule subset combines both prospective and retrospective data given the difficulty of discerning the two in some articles. The antibody subset combines all prospective data from internal studies in which SIE was applied to design novel mutants. As described earlier, SIE has also been tested extensively in community studies for small-molecule binding [SAMPL-1/3/4 (Sulea et al., 2012; Hogues et al., 2014), CSAR-2010/13/14 (Sulea et al., 2011; Hogues et al., 2016), D3R-GC2 (Hogues et al., 2018b)] as well as in-house studies of relative binding affinities for antibodies [SiPMAB (Sulea et al., 2016)]. From these studies, 975 and 212 data points were supplemented for small molecules and antibodies, respectively (Supplementary Tables S5-S6). To avoid skewing analyses towards these benchmark studies, these large datasets were treated separately.

**FIGURE 1:**
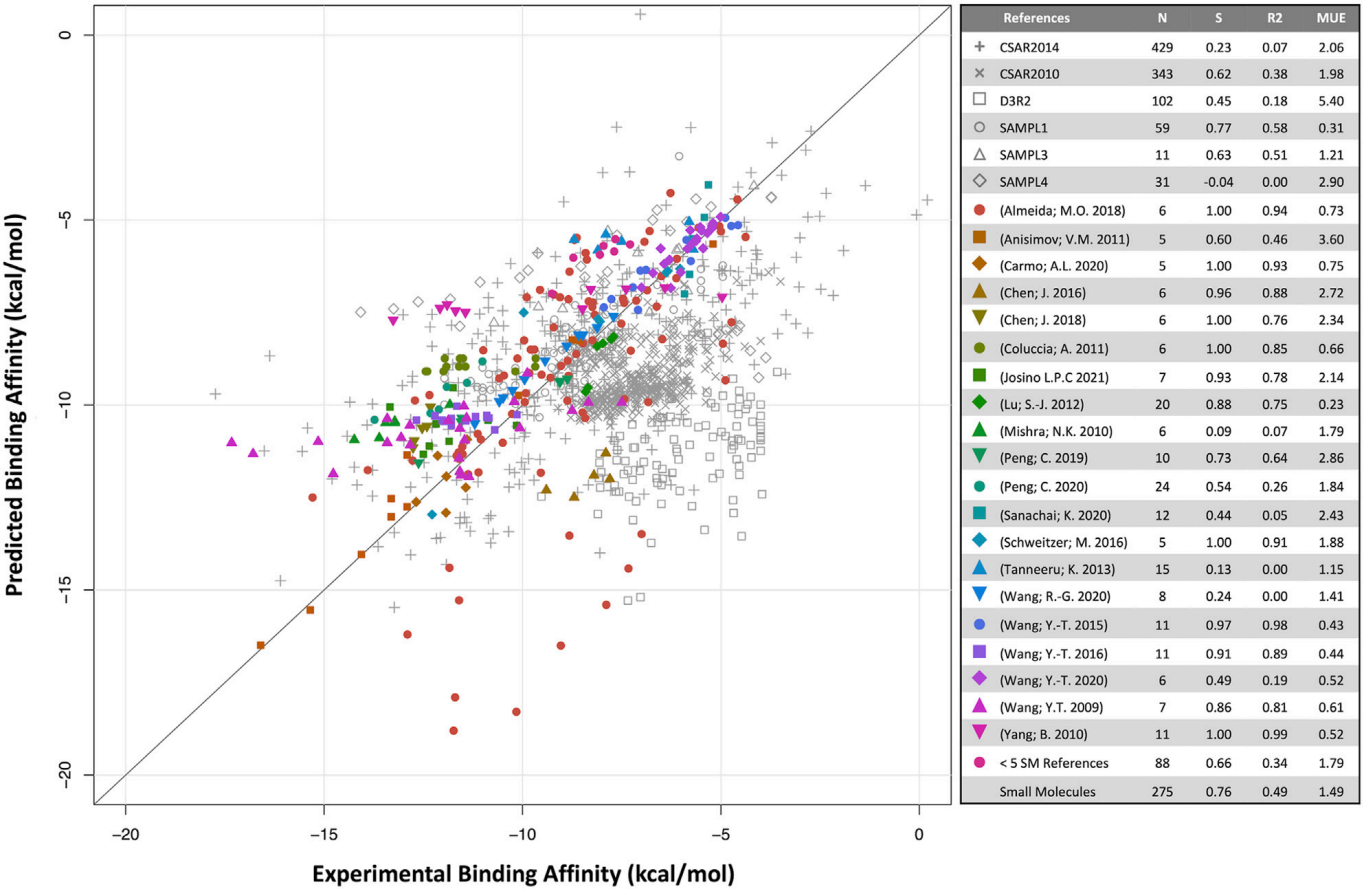
Predicted versus experimental absolute binding affinities for small-molecules. Data from external articles (colored symbols) and community-wide challenges (gray symbols). The “Small Molecules” set combines all data from external articles. Number of compounds (N), Spearman rank-order correlation coefficient (S), Pearson correlation coefficient (*R* ^2^) and mean unsigned error (MUE, kcal/mol) are listed for each set.

**FIGURE 2:**
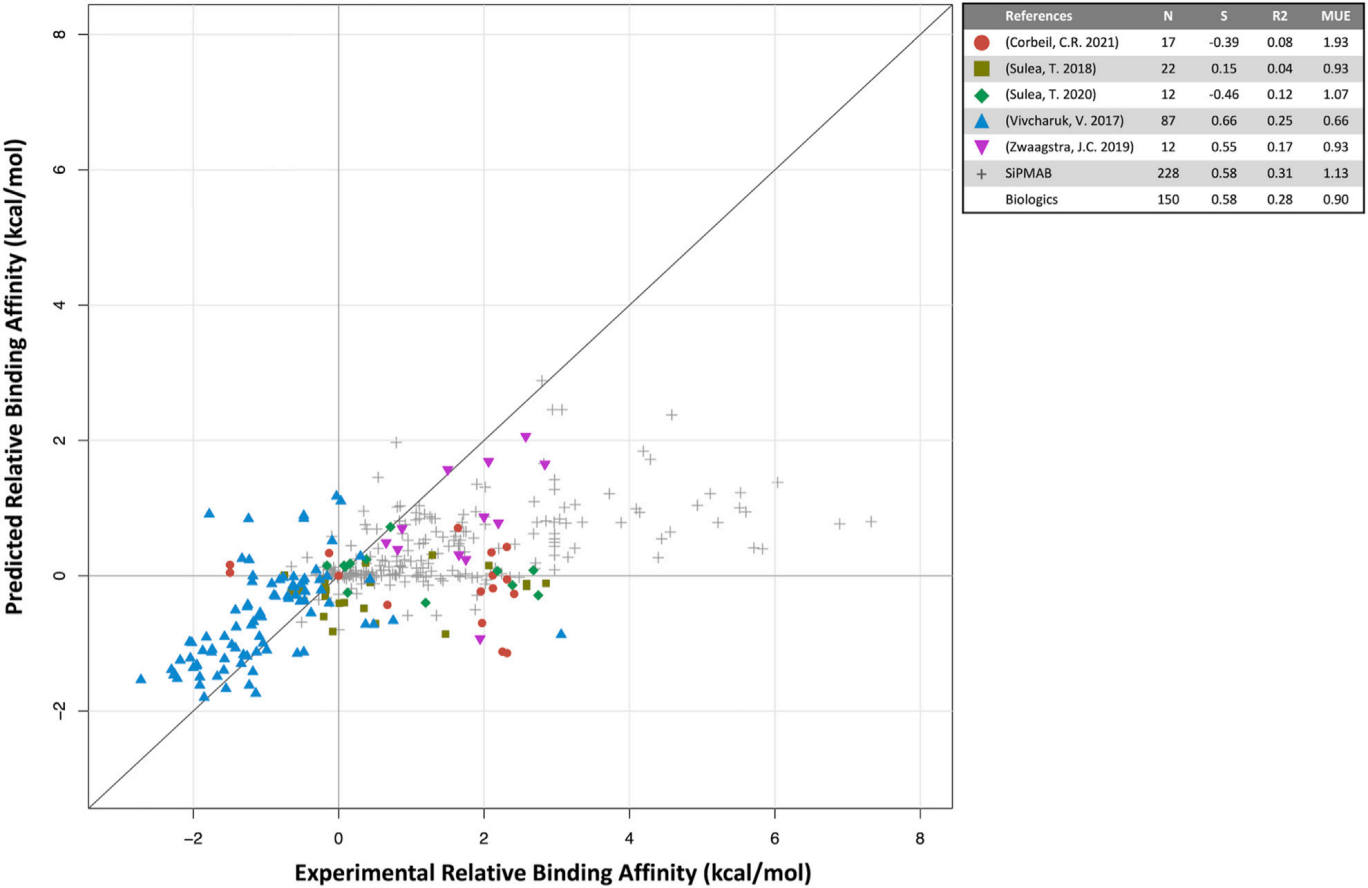
Predicted versus experimental relative binding affinities for biologics. Data from prospective studies (colored symbols) and in-house SiPMAB set (gray symbols). The “Biologics” set combines all data from literature. Statistical parameters as in Figure 1.

Predicted binding affinities were plotted against experimental data for small molecules (Figure 1) and biologics (Figure 2). Overall, SIE performs well on the published data for 275 small molecules, achieving good ranking (S of 0.76) and MUE of 1.49 kcal/mol. Both values are close to the published results for SIE on the training set (S of 0.79; MUE of 1.38 kcal/mol). Out of the 65 articles, only 10 of them contained targets present in the SIE training set (Supplementary Table S7). Individual correlations in most systems are reasonable, demonstrating transferability of SIE across multiple targets, even for those not part of the training set.

Aside from the overall binding affinities, analysis of the individual components of the SIE score can provide insights into the nature of the binding interactions. In a study of a Pan-BCR-ABL kinase inhibitor, the SIE components of the interaction of the inhibitor with the native and fourteen mutant BCR-ABL kinases revealed the relative importance of the electrostatic and van der Waals contributions to binding for the various mutants (Tanneeru and Guruprasad, 2013). SIE has also been used for virtual alanine scanning, systematically replacing selected residues with alanine and recomputing the predicted binding affinity. For example, it was used to identify which residues contribute the most to the binding of a peptide inhibitor to the MurA enzyme of *Pseudomonas aeruginosa* (Lima et al., 2017). Another application of SIE is in structural studies. In a study of putative binding modes of inhibitors to acetylcholinesterase, the SIE score in conjunction with qualitative structural analysis of the modeled structures was used to predict which binding mode was the most likely one (Galdeano et al., 2012). Drug resistance is a major concern in therapeutics. In a study of the drug resistance arising from HIV-Protease mutants, both SIE and MM-PBSA showed that the decrease in van der Waals interactions between the inhibitors and the protein was the driving force in conferring resistance (Wang et al., 2020). Polar interactions hardly contributed to drug resistance.

For biologics, 5 internal publications yielded 150 antibody mutants with both predicted and experimental binding affinities relative to the respective parental antibodies (Vivcharuk et al., 2017; Sulea et al., 2018; Zwaagstra et al., 2019; Sulea et al., 2020; Corbeil et al., 2021). When compared to the published results for small molecules, the quantitative prediction of relative binding affinity for antibodies is not as successful (S = 0.58, MUE = 0.90 kcal/mol). The overall correlation is mainly driven by three studies, Vivcharuk et al. (2017); Sulea et al. (2018); Zwaagstra et al. (2019) in which SIE was used to predict binding affinity changes mostly for single mutants relative to a parental antibody. They are similar in spirit to the SIE benchmark study on the SiPMAB set (Sulea et al., 2016), hence their comparable performances (Figure 2). The other two studies required introduction of additional degrees of freedom in the modeling approach, which may explain their poorer correlations. In Sulea et al. (2020) SIE was used to design mutants that selectively bound in the acidic tumor microenvironment, which necessitated predicting binding affinities for both neutral and acidic pH, which may have compounded prediction errors. Despite the low correlation observed, these predictions still proved useful (Section 4.3). In Corbeil et al. (2021) a mutational engineering endeavor was undertaken by attempting to redesign the entire CDR H3 loop of an antibody. The requirement of predicting the protein loop backbone conformation significantly increased affinity prediction errors. Protein flexibility and solvation are some of the areas which may require further development for improving predictions of relative protein-protein binding affinities.

## 6 Perspectives and conclusion

As with any scoring function, there is room for further improvement. A key component of the SIE scoring function is its solvation model, which has evolved in sophistication over time. Further refinement of the FiSH solvation model continues to be an active area of development. The original FiSH model was parameterized on small organic molecules. A possible refinement for applications to protein-protein interactions would be fine-tuning the parameters such as the born radii using molecular dynamics simulations of amino acids and short peptides with explicit water molecules as a reference. The scoring function also lacks an explicit conformational entropy term. Currently, an overall scaling factor is meant to capture entropy-enthalpy compensation, which can account for overall trends but is incapable of reproducing more granular details of entropic contributions to the binding free energy. Empirical entropic terms such as found, for example, in the FoldEF scoring function could be added. The conformational sampling as a by-product of the different methods used in the consensus approach does address flexibility to some extent, which may explain some of its improved performance.

Applications of machine learning and AI have exploded across almost all disciplines. The use of AI together with physics-based methods is a powerful combination. As discussed above, consensus scoring has been key in enhancing the robustness of ADAPT in antibody design. The incorporation of one or more AI-derived scoring functions (Li et al., 2021) as part of the consensus score could provide complementary information not well captured in the current physics-based functions. AI tools could also improve the SIE scoring function itself by optimizing the parameters in its solvation model. Moreover, since SIE is dependent on a 3D structure of the systems of interest, the increasing capability of AI structure prediction methods (Jumper et al., 2021; Akdel et al., 2022; Bryant et al., 2022) will have a collateral benefit in broadening the scope of molecular targets that SIE and other physics-based scoring functions can be applied to.

Despite its present limitations, SIE has been successfully applied across multiple systems by various research groups. Although initially developed for small-molecule affinity prediction, the literature we have surveyed highlighted its versatility as demonstrated in the variety of applications through the years that have ranged from small-molecule docking and virtual screening to the design of biologics. This wide applicability is remarkable given the parsimonious number of fitting parameters in the original calibration of the SIE scoring function as well as being relatively computationally inexpensive.
