# Supplementary material for: Solvated interaction energy: from small-molecule to antibody drug design
Source: Front Mol Biosci. 2023 Jun 7;10:1210576. doi: 10.3389/fmolb.2023.1210576 (PMC10282643; doi:10.3389/fmolb.2023.1210576)
Supplement: Supplementary file 1 [file DataSheet1.PDF]

Supporting information
for

Solvated Interaction Energy: From Small-Molecule to Antibody Drug Design

*Enrico O. Purisima, Christopher R. Corbeil, Francis Gaudreault, Wanlei Wei, Christophe Deprez, and Traian Sulea**

Human Health Therapeutics Research Centre, National Research Council Canada, 6100 Royalmount Avenue, Montreal, QC, Canada H4P 2R2

*Corresponding author: traian.sulea@nrc-cnrc.gc.ca.

LEGENDS:

Figure S1. Citations of key SIE papers by country.

Table S1. List of 377 distinct citations of 15 key methodological papers on SIE.

Table S2. List of 50 of the 377 citations (13%) for a different scoring function, called GBVI/WSA, calibrated on the SIE training dataset.

Table S3. Subset of 275 data points for small-molecule complexes collected from 65 articles.

Table S4. Subset of 150 data points for antibody complexes collected from 5 articles.

Table S5. Subset of 975 data points supplemented for small-molecule complexes from community-wide studies.

Table S6. Subset of 212 data points supplemented for antibody complexes from community-wide studies.

Table S7. References to 10 studies containing targets present in the SIE training set.

Note: Tables S1-S7 are provided in a separate Microsoft Excel file.

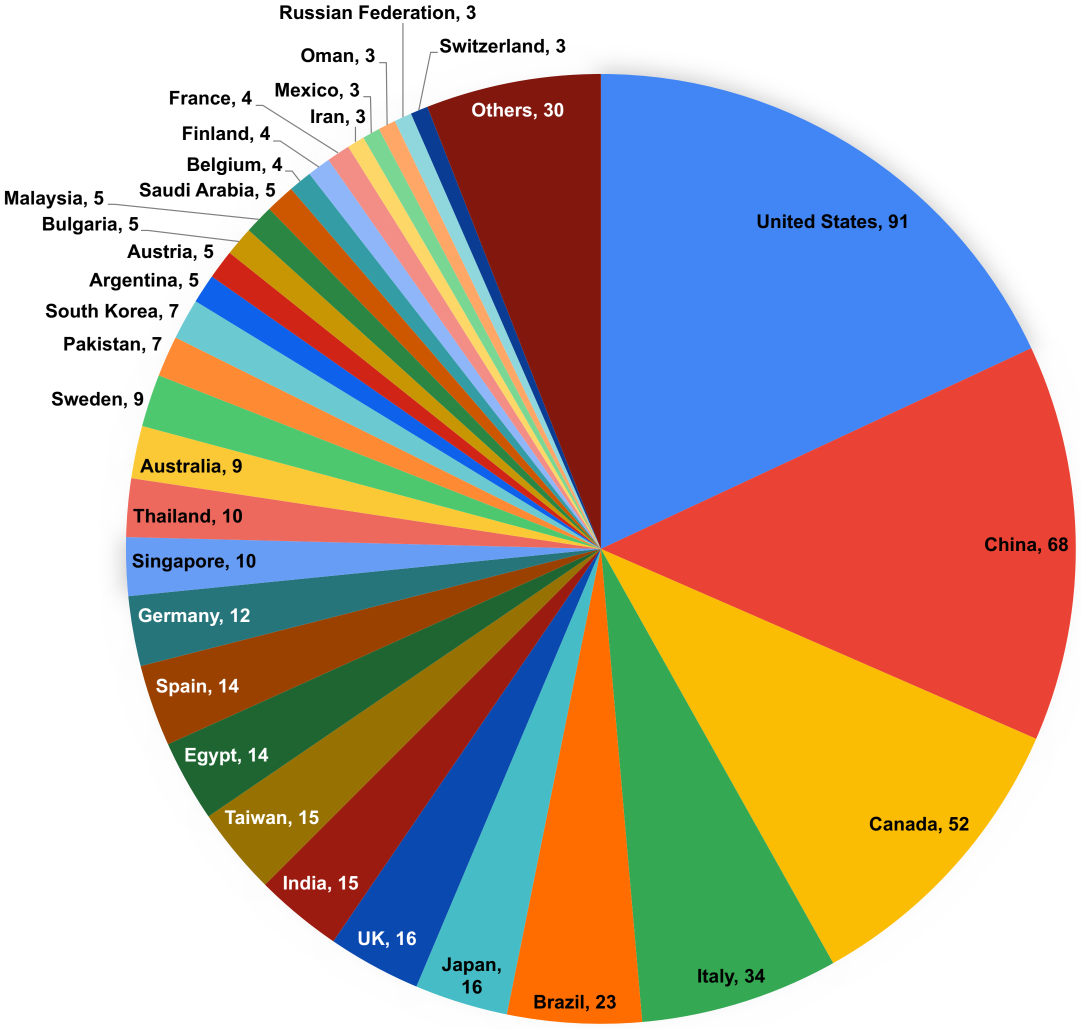

Russian Federation, 3
Switzerland, 3
Oman, 3
Mexico, 3
France, 4
Iran, 3
Others, 30
Finland, 4
Belgium, 4
Saudi Arabia, 5
Malaysia, 5
Bulgaria, 5
Austria, 5
Argentina, 5
South Korea, 7
Pakistan, 7
Sweden, 9
Australia, 9
Thailand, 10
Singapore, 10
Germany, 12
Spain, 14
Egypt, 14
Taiwan, 15
India, 15
UK, 16
Japan, 16
Brazil, 23
Italy, 34
Canada, 52
China, 68
United States, 91

Figure S1
